# Health Communication and Inequalities in Primary Care Access during the COVID-19 Pandemic among Ethnic Minorities in the United Kingdom: Lived Experiences and Recommendations

**DOI:** 10.3390/ijerph192215166

**Published:** 2022-11-17

**Authors:** Winifred Ekezie, Akilah Maxwell, Margaret Byron, Barbara Czyznikowska, Idil Osman, Katie Moylan, Sarah Gong, Manish Pareek

**Affiliations:** 1 Diabetes Research Centre, College of Life Sciences, University of Leicester, Leicester LE5 4PW, UK; 2 Centre for Ethnic Health Research, University of Leicester, Leicester LE5 4PW, UK; 3 School of Media, Communication and Sociology, University of Leicester, Leicester LE1 7RH, UK; 4 School of Geography, Geology and the Environment, University of Leicester, Leicester LE1 7RH, UK; 5 Department of Respiratory Sciences, University of Leicester, Leicester LE1 9HN, UK; 6 National Institute for Health Research, Leicester Biomedical Research Centre, Leicester LE3 9QP, UK

**Keywords:** COVID-19, ethnic minorities, communication, misinformation, public health, health inequality, qualitative, United Kingdom

## Abstract

Health Communication is critical in the context of public health and this was highlighted during the COVID-19 pandemic. Ethnic minority groups were significantly impacted during the pandemic; however, communication and information available to them were reported to be insufficient. This study explored the health information communication amongst ethnic communities in relation to their experiences with primary health care services during the COVID-19 pandemic. The research used qualitative methodology using focus groups and semi-structured interviews with community members and leaders from three ethnic minority communities (African-Caribbean, Somali and South Asian) in Leicester, United Kingdom. The interviews were audio recorded, transcribed, and open-coded. Rigour was determined through methodological coherence, appropriate and sufficient sampling, and iterative data collection and analysis. Six focus groups and interviews were conducted with 42 participants. Four overarching themes were identified related to health communication, experiences, services and community recommendations to improve primary care communication. To address primary care inequalities effectively and improve future health communication strategies, experiences from the pandemic should be reflected upon, and positive initiatives infused into the healthcare strategies, especially for ethnic minority communities.

## 1. Introduction

Communication is critical in the context of public health and outbreaks, and the COVID-19 pandemic has brought this into stark view with issues relating to fear and the origin of the virus. Initially, communication on COVID-19 started from the global level before scaling down to local contexts. The World Health Organisation (WHO) guided national governments in communicating COVID-19 risks and safety precautions. In the United Kingdom (UK), at the national level, the communication strategy was adapted from previous flu outbreak experiences and focused primarily on disseminating health-related information to control the spread of the virus to the public [1]. The UK-level communication strategy was divided according to pandemic response phases and included: a forced lockdown policy, stay-at-home order except for only limited exercise, medical reasons, or essential shopping with social distancing of two meters; in addition, people who were vulnerable and those with serious medical problems were shielded and told not to leave their homes [2]. Hence, these restrictions significantly reduced the frequency of face-to-face primary care appointments, consultations and communication.

Based on global and national trends of COVID-19 impacts on ethnic minority groups, health communication was a crucial factor in managing the crisis. As such, more evidence towards understanding how information during the COVID-19 pandemic phase circulated and how the information impacted actions among different communities is crucial. Due to the unfamiliarity of the disease and uncertainty of the actions and impact of the virus, the rapidly evolving communication, which often changed over short periods, produced conflicting messages resulting in a breakdown in public trust in the government and healthcare services, especially among ethnic minority populations [3,4]. Hence, combating the pandemic challenged and revealed communication frictions between scientists, political parties, health providers and the general public. This potentially exacerbated existing societal inequalities, especially among ethnic minority populations [5]. Furthermore, public restrictions significantly impacted access to primary healthcare communication, with a more significant impact observed among ethnic minority groups—who were already significantly more affected by COVID-19.

Factors that increase the risk of COVID-19 infection, hospitalisation, and death include age, pre-existing health conditions, ethnicity, household size, and occupation [6,7]. In the UK and United States (US), these biomedical risk factors and social determinants contributed to wider health disparities and were associated with inequalities that resulted in higher COVID-19 outcomes among ethnic minority populations [8,9,10,11]. Multiple factors, including socio-demographic factors, influenced individual health and gave rise to these health inequalities. Other factors that increased the vulnerability of ethnic minorities to COVID-19 included geography, occupational exposure, family circumstances, and economic disparities [7,12,13]. Hence, health inequalities can be defined as systematic, and potentially remediable differences in one or more aspects of health across populations or population groups defined socially, economically, demographically, or geographically [14,15]. Additionally, living and working practices were believed to increase the chances of infection and death [8,16,17,18].

Generally, primary healthcare access inequalities have been observed across the UK population [19,20]. Primary care services (general practices) are well positioned to reduce health inequalities through clinical care, patient advocacy, community engagement and influencing broader political agenda [19,20,21,22,23]. Furthermore, good clinical practice involves GPs being aware of demographic data pertinent to health inequalities, e.g., potentially vulnerable groups, and actively seeking to address them [21,22,24]. However, despite the disproportional impact of COVID-19 on ethnic minority communities, many patients still could not access their usual direct care from a healthcare professional, and although technology improved accessibility, it was not convenient for all population groups, for instance, groups who experienced communication and language barriers [5]. This study, therefore, aims to explore the health information communication amongst ethnic communities in relation to their experiences with primary health care services during the COVID-19 pandemic. In addition, the community initiatives implemented and recommendations for future actions and interventions are presented.

## 2. Materials and Methods

### 2.1. Study Design

The qualitative study comprised focus group discussions with community members and semi-structured interviews with community leaders from three ethnic minority groups: Asian, Somali and African-Caribbean.

### 2.2. Study Setting and Participants

The study was conducted with selected majority ethnic minority communities in Leicester, United Kingdom, between May and September 2021. Ethnic minorities within the context of this study were all ethnic groups except the White British group. Leicester is a multicultural city with 51% of the population identifying as ethnic minorities and was also the first area in the UK placed under local lockdown in response to spikes in COVID-19 cases between June and August 2020 [25]. Recruitment of participants was done through existing Patient and Public Involvement (PPI) networks, social media, radio broadcasts and snowballing. Participants were also identified using purposive sampling based on personal interest in the study and individual roles and responsibilities. Six focus groups were conducted, two group discussions with 6–8 participants in each of the three ethnic groups, while individual interviews were held with two community leaders from each group (total = 42).

The focus group and interviews were conducted by two authors (BC and AM) who have extensive experience working with ethnic communities. Topic guides used are included in the Supplementary S1. The focus group structure considered cultural factors (e.g., single-gendered groups) and availability. One Somali group was split into smaller focus groups of four participants to accommodate both genders, and one African-Caribbean group was divided into two smaller groups to accommodate participant schedules. All focus groups were conducted virtually using the Zoom platform to adhere to COVID-19 social distance guidelines and national protocols, except for the Somali group discussions, which were conducted in a community centre to provide language support with assigned translators based on participant preferences. The individual interviews were held in public spaces and socially distant to ensure the safety of respondents and interviewees. Eligible participants were notified to seek their interest and availability approximately two weeks in advance, and consent was obtained from each participant before data collection. Ethical approval for the study was received from the University of Leicester Medicine and Biological Sciences Research Ethics Committee.

### 2.3. Data Collection and Analysis

The interview topic guide covered topics on COVID-19 information sources, perception and engagement, the impact of the pandemic, and health service accessibility (see Supplementary S1). Data collection was conducted between June and September 2021 using online video platforms and in-person meetings. Interviews and discussions were conducted in English, and translation into Somali language was also provided. Group discussion lasted approximately 90 mins, and interviews lasted approximately 60 mins. Audio recordings were transcribed verbatim directly to English by AM. Afterwards, two other researchers (WE and IO) verified each transcript for quality, consistency, and accuracy. An inductive thematic approach was used to analyse the data using the QSR International Pty Ltd.Nvivo 12.0 software (released in March 2020), which was sourced through the University of Leicester’s Software Centre. The analysis followed the six-stage process proposed by Braun and Clarke: familiarisation with the data, generating initial codes, searching for themes, reviewing initial themes, defining and naming themes, and producing the report [26]. Double coding of transcripts was conducted by AM and WE to enhance the transparency, validity, consistency, and applicability of identified themes and subthemes.

## 3. Results

**Figure 1:**
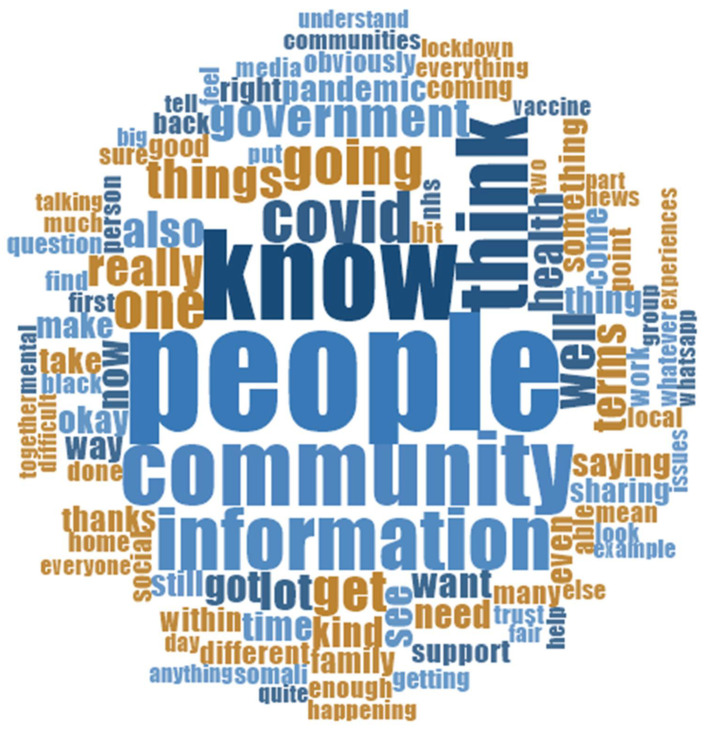
Word cloud of 100 most commonly used words by study participants.

A total of 42 individuals, 14 participants from the three ethnic groups (African-Caribbean, Somali and South Asian), participated in the study. Focus groups were conducted with 36 community members (12 from each ethnic group) and one-to-one interviews with six community leaders (two from each ethnic group). There were more similarities compared to dissimilarities in the COVID-19 pandemic and communication experiences across the different ethnic groups; however, some ethnic and cultural practice differences were influenced by factors such as household dynamics and language. Figure 1 shows a word cloud of the 100 most common words used by participants. The top five words “people, know, community, think, information, COVID” effectively summaries the key emphasis on the need to reflect and understand people and communities when developing COVID-19 information communication. The word cloud also highlights participants’ interest in government actions, trust, information sharing, family, social and health issues. The experiences across the three ethnic groups were explored, and thematic analysis of the data revealed four overarching themes and 14 corresponding subthemes (Table 1).

### 3.1. Health Communication

Sources of health information during the pandemic varied by type, medium, and individual preferences. Community representatives explained that health providers generally ignored cultural factors when communicating and attending to patients from ethnic minority backgrounds. The overlooked issues included a lack of language support, recognition of cultural dynamics and communal practices, and these gaps gave rise to conflicting information and confusion.

#### 3.1.1. Communication Source and Preference

During the pandemic, the public relied predominantly on communication from the central government more than their primary healthcare services. As a result, participants stated there was excessive information, a mix of approved government guidelines, cultural practices and remedies to maintain health, unreliable information, and myths about COVID-19. They reported directly seeking COVID-19 health information from various sources, including traditional mainstream news media channels, social media, word of mouth and the internet. Traditional media options included news broadcasts, updates and discussions on television and radio, such as BBC News and radio stations, while commonly used social media sources were Facebook, Twitter, TikTok and WhatsApp. Some individuals reported also accessing health information from websites of official health organisations, such as the NHS, WHO, and the Centre for Disease Control (CDC). In addition, some participants received information through communications in leaflets and letters circulated by their local authorities and voluntary sector organisations. A few participants also sourced information using mobile applications (apps) such as the COVID symptom and NHS COVID apps. In addition to UK-based local and national updates, some participants also followed the information communicated in different parts of the world, particularly from their countries of origin. One reason for seeking information from a wide variety of sources was that the news they needed was sometimes inconsistent, insufficient, and arrived late, as described by one African-Caribbean participant:

*(information sources include) the World Health Organization, and so we’ve got family in the Caribbean, so we have access to some of the things they’ve been saying. And it’s looking at, like, why are they saying the things that they are saying? So, over here, we kept saying stuff like “common sense”, and it’s like, “no, it’s not a valid reason to do anything”. But other places were saying things like, “we’re doing this because the research currently says this”. So I was like, “Ok, that I can take on”.—**African-Caribbean***

Considering most information was in digital formats, experiences with communication engagement were further complicated by other social factors such as digital poverty and low confidence in using devices. These issues posed particular challenges among the elderly, some disadvantaged groups and individuals with poor English skills, who therefore had to rely on others, especially family members, to provide them with needed information. For families living in different locations, the responsibility of sharing information was reported to be really difficult. The challenging experience was described by a participant who also highlighted the challenges felt by different groups:

*I think if you’re competent with the digital way of doing things, you can access a lot of things. Sometimes it’s a bit tucked away. It’s in like the Gov sites, or it’s in the local authority sites, or one of the sites I’ve used… but it’s knowing where to look… But if you’re more vulnerable, like if you’ve got special needs, or if you’ve got a disability or language barriers, then accessibility is quite tough… I’ve noticed in my kind of community or my elderly relatives where they’ve struggled, is because they don’t know how to use computers or internet. So they all rely upon other people who are a bit more savvy with that…—**South Asian***

Participants shared that delays in communication heightened overall stress and fear. Consequently, some people avoided news channels or limited the frequency at which they consumed information updates. Nevertheless, community members preferred communication from someone within their community besides their primary care GPs. One African-Caribbean participant explained that this was because such people had the best interest of their community at heart and would provide more trustworthy information:

*… the main thing that worked for me was to have somebody who I knew who is in the medical profession, who I knew, advocated for black people, for them to say, this is something that you should do… I was fortunate because I knew those people. But if those people were, because it’s not just GPs—like any other doctor, I probably would have thought, Yeah, well, you’re gonna say that anyway, but because it’s people I knew were actively working for the benefit of black people… I knew that they would do… medical experts who can vouch for the efficacy of drugs.—**African-Caribbean***

#### 3.1.2. Vaccination Communication

Participants complained about the lack of clarity on the vaccination and the way poor communication impacted people’s perception and attitude towards the COVID-19 vaccines. In addition, a participant elaborated that concerns and controversy around the speed of development propagated the circulation of negative information amongst the community:

*A lot of people were just saying, government is just doing this, forcing us. But there isn’t a clear picture in terms of what is the vaccination? Everybody knows it is for COVID, but what does it have? Does it have any animal products? Anything that will have a lasting effect on their health, people with medical conditions, health conditions, or underlying issues? There was no clarity on any of those. Just simple sort of messages that was getting vaccinated, get vaccinated, it is safe … Then people started to question because a report came out about blood clots and stuff like that. Then people say see we told you … and what happens afterwards, and people would believe what was a myth was now the reality.—**South Asian***

Regarding delivery of the information, participants further highlighted that not seeing “relatable people” sharing accurate health information about the vaccines encouraged more mis- and disinformation to circulate. However, as more information became available to the communities, vaccination uptake trends increased for various reasons. Community leaders explained that local campaigns were carried out in collaboration with healthcare providers who were members of their community. In addition, NHS-controlled vaccination centres were made available in various community settings, and more were provided in the hardest-hit areas of ethnic communities. Participants stated that this approach increased accessibility to vaccines across all age groups.

*…they did a campaign, a huge campaign for vaccination. They were explaining to people and they were engaging … I would like to see how they campaigned for the vaccination and involved everyone … They didn’t do that for COVID-19 vaccination… they will talk about COVID-19 and wash your hands and these things, but that is the things that people could see … people also could read about the vaccination against COVID-19 … they spent huge money in order to persuade people to have the vaccination.—**Somali***

#### 3.1.3. Language Barrier

The most significant barrier to communication across all the communities was language. This included the ability to speak English and understand information when communicating with health providers, especially during remote consultations. The challenges were reported more among the Asian and Somali communities, and one Somali representative described their experience from GP interactions:

*COVID-19 was a strange period and shows the differences between communities and the impact the problem has on different communities. How can somebody who doesn’t speak the language explain what they have when everything is closed? You can’t come to the GP, the only activity is phone communication. On telephone communication, I can’t explain to you what I have, so that is the problem.—**Somali***

Many participants, particularly among the Somali and South Asian communities, reported that before the pandemic crisis, they usually served as translators for non-English speaking family members and friends when meeting with doctors and other service providers. However, during the pandemic, access to and acceptance of information were further impacted by the cultural practices of specific groups. Cultural influences on information acceptance were widespread among the elderly who spoke little English, had stronger cultural beliefs and practices and relied significantly on information from local leaders. These were also reported more by the Somali and South Asian communities, and participants stated that the combination of these two factors, language translation and cultural practices, influenced the acceptance of COVID-19-related information even when family and friends acted as interpreters. Consequently, some participants reported some cultural tensions and collusion experienced across different generations—young vs old. It was therefore specified that the identity of a person providing COVID-19 information often determined the way in which the older community members would accept it. This experience was described by a second-generation South Asian community member who explained their experiences of translating to older family members:

*“… you might have somebody who is very educated and is dealing with a parent or a grandparent who is not familiar with the words … [or] understand any of the jargon that’s going on. They are waiting for you to interpret it … if you are young, they are not really believing you because they are wiser [and] they have more [life] experiences … even if you have explained it to them they still would not believe you … it does not mean they will agree.”—**South Asian***

Participants proclaimed that the lack of clarity in the national guidelines for ethnic minorities and the presentation of this in formats they can use contributed to the mistrust in communication from official channels. Furthermore, the limited guidance from primary healthcare providers at the beginning of the pandemic spurred people to seek information elsewhere, which often included myths, conspiracy theories and misinformation. As a result, some participants stated that COVID-19 myths and communication within their community often overlapped, and this was actively circulated during family and friends’ communication, particularly on Facebook, WhatsApp, or phone calls. Hence, some participants proactively avoided some social media platforms. One participant elaborated on this and described that not having community-tailored information encouraged the circulation of wrong information:

*As UK, they don’t address the specific communities … if people are new in this country and they don’t know where to get the information or something like that, then they are vulnerable and will take all the misinformation that they are getting through the internet.—**Somali***

#### 3.1.4. Community-Tailored Communication

Communities reported noticing several barriers to health communication, which had existed even before the pandemic. One participant stated that primary healthcare doctors did not adequately meet the needs of the patients and that going directly to secondary care (hospitals) was better.

*GPs are the first contact point and yet they are not even meeting the basic support service like sorting out appointment with the client and listening to the client. Even not giving enough time, they feel they don’t have enough time. Because of language barrier is my take or they don’t understand the real issue … hospital is better, secondary service is better … Because secondary service, you are more likely to get attention—**Somali***

Some participants also expressed that because ethnic minority communities were often pushed aside and left behind in terms of information in general, local community organisations decided to take the responsibility to provide needed information so community members would not feel lost and isolated. Community leaders and members, therefore, took the initiative to gather information from trusted websites and curate them for their communities. In addition, they also partnered with health practitioners who were members of their community to debunk myths and misinformation. A representative community leader describes the approach used by members of the African-Caribbean community:

*African heritage community is one of those communities that often gets pushed aside and also get left behind in terms of information. So that we had to shape the information and get it out there to the community so that they didn’t feel lost and isolated, but felt that they had a way of communicating with each other … using a community radio is informing individuals about what the messages are for COVID and physically inviting the Director of Public Health onto that show … to bring this live program to try and debunk some stories out there.—**African-Caribbean***

### 3.2. Health Experience and Impact

The pandemic affected communities directly (COVID-19 infection and death) or indirectly, personally or across their communities and households. The changes in access to public services also impacted the care demands and responsibilities, and a significant outcome observed was the effect it had on people’s mental health.

#### 3.2.1. General Health Experiences

Participants stated that COVID-19 had far-reaching health implications, directly and indirectly, across their communities. Direct impacts were experienced by those who got infected with the COVID-19 virus. Although only a few of the sampled participants had been positive, nearly all participants had someone close to them who had been directly affected. As described by one participant, having COVID-19 impacted both the infected person and their community at large:

*I was the victim of COVID itself. I was in hospital for six, seven days, on oxygen and NHS did a good job for me, it was at the time when it was peak time. So it became quite stressful at the time, for me, for my family, for relatives and everybody … I think they were all concerned for each other.—**South Asian***

The broader indirect health impacts of the pandemic increased anxiety and loneliness primarily resulting from the fear of contracting and passing on COVID-19 infection primarily to family, as described below:

*For family, a lot of people are so frightened of actually coming anywhere near you, which I know for many has led to increased anxiety, what they may pass on, and having to live with those consequences, the guilt equally for the families can lead to quite a lot of loneliness.—**African-Caribbean***

Consequently, there was a reported rise in mental health issues and chronic diseases, including diabetes and hypertension. The combination of these health issues was stated to have increased the risk of worse outcomes if affected individuals also got infected with COVID-19. Participants also explained that the rise in depression and anxiety in their communities was more prominent amongst younger people. The escalation in mental health concerns encouraged ethnic minorities to rethink approaches to mental health, and some participants indicated that due to their experiences, the views of communities about health had changed to some extent. For example, one Somali community member stated that they relied on their Imams (religious leaders) for support:

*Diabetes has increased, the mental health issues have increased, now we need to make sure after COVID, how we can deal with those impacts because people always think [ing] of death and what it costs and life … a lot of frictions and the Imams were there to solve the domestic matters.—**Somali***

However, despite the health challenges, some participants reported that many of their community members were still apprehensive about seeing a GP or visiting a primary health centre or hospital for any health procedure because of the fear of dying and not returning home. One participant explained that such actions resulted in the late presentation of some conditions to health centres which often worsened the outcomes:

*And then people fear bringing loved ones for medical attention when they need it. So what we see is a lot of delayed diagnoses of people trying to weigh up whether or not the more at risk of fatal cancers and dementia has become deteriorated an awful lot.—**African-Caribbean***

#### 3.2.2. Caring Responsibilities

Having COVID-19 and/or supporting those who were ill with COVID-19, but with limited community support and interaction, increased the caring responsibilities of some community members. Some participants stated that they took on most of the responsibility for caring for family members with chronic health conditions with minimal social support from their GPs and the NHS. They stated that although GP surgeries had carer registers, they had to wait for priority services. For instance, they were not provided the same priority access to the COVID-19 vaccine as the people they were caring for, despite being a risk to them if they got infected. Some participants also criticised the general lack of support and unclear communication for managing COVID-19 within a family unit and the burden of acting as carers for vulnerable and elderly family members. This experience was illustrated by participants who shared frustration while undertaking their caring responsibilities.

*…before lockdown, carers had already been isolated. And because of the carer’s commitment, they were too busy caring for their loved ones they can’t get out and they are very much dependent on the information that was signposting. And once the COVID started, lots of their partners have been shielded … information they receive from the government department or from the council … I have couple friends who rang me to say they could not understand the shielding element, it wasn’t properly explained, like what the shielding was. And to make matters worse, because of the lockdown and all that we have really been isolated… And added difficulties was communication with the medical professionals … you have to wait ages … And that makes things a lot difficult.—**South Asian***

A major communication role most participants mentioned taking on as carers was the responsibility of being a translator. Some expressed that those caring for their family members also took on the role of interpreters during remote consultations with their GPs. A few of them stated that translating was a new task they took on during the pandemic, which proved quite challenging.

*But in terms of my mother, she, at her age has many needs to see a doctor. And it becomes very difficult … she’s also suffering from a hearing problem. So I now become a translator… I’ve got to translate from my mother to this doctor. And then vice versa, because the doctor isn’t there to probe and to check. That becomes quite difficult—**African-Caribbean***

Consequently, several people with caring responsibilities reported feeling overwhelmed by caring for COVID-19-infected family members, juggling family responsibilities and facing isolation, and that the accumulation of these factors affected their mental health. Overall, such experience was reported more by South Asian participants, who, compared to the other ethnic groups, often lived in large and multi-generational households.

*… a lot of carers before COVID, they do go on to develop either mental health or physical conditions. So this is kind of exaggerated more as into the mental health, depression, anxiety.—**South Asian***

#### 3.2.3. Mental Health

The common secondary health challenge arising from the pandemic was mental health-related issues. Several participants reiterated the increase in mental health issues directly linked to the pandemic experiences, especially among young people. Across all three community groups, there were reports of significant exacerbation of both pre-existing and new mental health challenges, in addition to the loneliness, fear of infection, death and caring responsibilities, and these challenges included anxiety, depression, stress, concerning coping mechanism with hints of suicide, and family problems such as domestic abuse. Related experiences from all three ethnic groups are shown below:

*… mental health (has) gone through the roof. I don’t think it’s the loneliness, but mental health and coping mechanisms, have overwhelmed both … we’ve had a youngster eight-year-old want to commit suicide… (said) I don’t mean it from the sense of “I’m going to commit suicide”, just words they are actively attempting.—**African-Caribbean***

*… mental health has increased and going forward, through young people, elders; and that is at the moment, I think, is the priority in our community to really deal with anxiety levels, and you know, stress and losing finances.—**Somali***

*I’ve seen a high rate of anxiety, depression, and domestic abuse as a result. So they’re the kind of key issues that have arisen because of COVID.—**South Asian***

Participants also indicated that some anxieties were reactions to the negative information aired on communication outlets and social media. This included information on high infection and death tolls globally and among ethnic minorities. Hence, some participants stated they decided to “switch off from COVID” to normalise their lives for their “sanity”. One participant stated that the information from social media caused them more trauma, so they disengaged from the wider community and global information. Instead, they received updates from their immediate social network.

*I used to do Facebook quite a bit. But I had to put that away. Because I was finding that it was bringing more trauma into my life … had to leave that alone and just work with the small amount of people in my family or my extended group of people outside.—**African-Caribbean***

Despite the rise in mental health issues, many participants expressed lacking access to mental health services and practitioners. Participants also shared their concerns about inadequate support available to effectively manage COVID-related mental health issues and emphasised that more support will be necessary to heal from the trauma. This sentiment was further explained by a South Asian community representative who expressed concern about the lack of mental health awareness among the community and health care support to address the issues:

*… this COVID, on the onset and still ongoing, is creating a lot of low level to medium-level mental health issues and it’s not being picked up and to get even the support out to the community … there are people who need support. There’s a huge gap.—**South Asian***

Among a few who had access to mental health support, such as those with caring responsibilities, participants stated that some people had declined to use those services due to the stigma associated with mental health within their community. This rejection of mental health services was also linked to the community’s lack of mental health awareness. For instance, those with concerns such as depression and anxiety were mostly advised to “pray and to ask God for help”; males were often told to “deal with it and go on” and were not encouraged to talk about their feelings. Hence, participants highlighted the need to improve mental health knowledge among community members:

*… if you are a carer or you have got identified mental health needs, there are support available through social services. But if a person has got stigma attached to service in the first place, they may not necessarily want to be caught contacting social services to access that support … there’s a level of education needed about social services.—**South Asian***

However, during the pandemic, there was a slight improvement in mental health acknowledgement and support acceptance, but religious leaders mostly provided this support. However, these options were also restricted due to the stay-at-home restriction order and closure of public spaces during the peak of the crisis, so communities adapted their approaches. One participant explained the way in which community leaders reach out to support their members:

*Imam also adds the mental health aspect because the Mosque [is] where the community comes together, where the problems were solved. Physically, persons would go there, talk to others. But the restrictions that people were confined in their own homes … They felt really overwhelmed … elders call the people who were vulnerable.—**Somali***

### 3.3. Health Service and Management

The way communities managed every aspect of their lives was influenced by accessibility, experiences with health services and communication. Despite the direct health challenges encountered, many community members hesitated or refused to go to healthcare centres for professional care for various reasons, such as distrust of health services.

#### 3.3.1. Health Service Access

The stay-at-home orders and health system factors, such as the overcrowding of health facilities due to high admission of COVID-19 patients, strained access to primary healthcare. Most interactions with primary health providers were conducted remotely, while face-to-face care was based on the urgency and vulnerability of the individual patient’s condition. One participant thought that the location also influenced the mode of service (face-to-face vs telephone):

*… with me being pregnant. I’ve been able to still have my midwife appointments, whereas other people within our community have not had that privilege, because their GP was not allowing that sort of interaction. I think it has to do with where they’re from, as well … my friends from London … everything was done over the phone … in Leicester, they were more open to seeing their patients face to face.—**Somali***

Participants further expressed general frustration in trying to access primary health services. They reported difficulty obtaining appointments to see a GP and long waiting times on the telephone for a remote consultation when the issues they had were considered “non-emergency”. These experiences further discouraged people from accessing primary health services and seeking direct health information and guidance from trusted medical sources.

*… initially, there wasn’t that much engagement (with trusted sources). It is difficult to actually get an appointment to see your GP … Unless you are very ill … during the pandemic, we should be able to get access to a GP more easily, but now it became more harder—it’s the opposite.—**South Asian***

With most of the services being provided online, several participants reported missing the personal connection achievable only during consulting face-to-face with their GPs, and the difficulty in booking appointments caused some frustrations and reluctance to bond with health service teams. From this experience, participants from all three communities hinted at the impact of the loss of connection with their healthcare provider and stated that having a family doctor who understood their personal needs helped ease the communication and personal connection barriers faced by appointment:

*… if I have an issue now, I’d have to call and then I’d have to describe … a photograph of it and send it off to them … I lose that personal connection. I want him to see it instead of sending off a photograph … that’s a massive change, a massive difference … It also prevented me from calling; whenever there is a problem, I put it off … instead of the usual thing is to go see my doctor, I can’t do that. And those problem becomes major issues.—**African-Caribbean***

*… it’s very difficult to get appointments with the GPs, and possible you can get is telephone consultation… create a kind of frustration. And we eventually become very reluctant to bond with the medical professionals because of frustration.—**South Asian***

*… NHS and GP, not really communicate well with the community. It’s very hard. People complain a lot about GP access, and making appointments. The reason being the way GP works may be tainted or you don’t have a family doctor or someone who knows well always you see a different person.—**Somali***

Participants also shared that there were some factors more particular to ethnic minority communities, which the government did not adequately consider in the pandemic communication, for instance, the high burden of specific conditions among some ethnic groups, e.g., diabetes among South Asian communities. Managing such long-term conditions was also challenging due to the closure of GP surgeries, limited appointments to see doctors and restrictions on hospital visits. One participant shared the despondency of many community members who had to rely mostly on self-care management, e.g., taking their medication based on past prescription recommendations:

*So any long-term illness people had or from previously, the doctor surgeries were closed … people have not even had a chance to talk to the doctor to get back to their original problems … (they were told) all you know, just continue to take your own medicine …. they were not able to go to the hospital because of the restrictions.—**South Asian***

Several participants expressed high levels of distrust in healthcare services. There was speculation that some GPs had used the COVID-19 crisis as an “excuse” not to provide face-to-face services; hence the care provided was inadequate and inappropriate for ethnic minority community needs. For example, a community leader believed that care provided to ethnic minority groups was not the same as that provided to those of White ethnicities, and the evidence was shown in the high death rates among ethnic minorities; a community member shared an opinion on the government being inconsiderate of people in the middle and lower class:

*… the treatment that some people got, especially the BAME community, was not up-to-date or was not the same as the other communities like the White community … many people who went into the hospitals passed away, and people are asking why … their mothers and fathers were at the hospitals… (they) couldn’t eat properly, nobody looked after them properly …—**Somali***

Despite the struggle to access health services, some participants stated they had been fortunate to have access to their GPs and healthcare team—e.g., pregnant women still having their midwife appointments—and they commended the efforts of these healthcare providers. A few participants then suggested that all staff working in the NHS, besides doctors and nurses, needed to be recognised for supporting and helping doctors and nurses in this challenging time:

*… to be fair, to the government and NHS, the NHS had done a good job … not talking about just the doctors and nurses, but all the others, staff admin, to the cleaners, to the people who deliver food to the table …. All this staff also had underlying issues, diabetes, blood pressure … still had to continue working.—**South Asian***

#### 3.3.2. Community Actions

Community leaders stated that at the start of the pandemic, they struggled to effectively share COVID-19 information with their members due to limited access to coherent government guidance. In addition, they stated that the communication in the English language only compounded the limited access to initial COVID-19 information. Hence, they proactively sought and approached local authorities, health services and GPs to provide resources in community languages. This was illustrated by a community organisation representative who explained their experience of accessing information and support during the pandemic:

*… when lockdown had started, we had very little information from the local authority … communications were very poor in terms of what the issues were and how to deal with COVID as individuals, as families and as the community centre … information was not in the correct language … targeting those communities that could not speak English.”—**South Asian***

Participants also stated that the community leaders and activists functioned as interpreters and coordinators and gathered government and NHS guidelines to translate for their community. Community leaders, including religious leaders, reported helping dispel myths and share information on vaccination processes. Informal communication systems were also used to discuss vaccination fears and ethical concerns; for instance, the concern of Muslims that the new drugs adhered to religious practices (i.e., Halal approved):

*… taking proactive steps to set up WhatsApp groups … sharing information nationally or locally, whatever comes through, whether it’s coming from NHS or government, or other scaremongering … we provided counter-narrative stories, genuine stories by getting the right information from the government, website and NHS.—**Somali***

To further bridge the communication gap and deter the spread of misinformation, community leaders stated they used virtual spaces such as online Zoom meetings to interact with other community members. The information provided during the public discussions included ways to stay safe, protect the larger community and support members who experienced mental and emotional stress. For example, one participant explained that some women in their community were supported through online video communication in creating healthy habits during the pandemic, as described below. Thus, the pandemic encouraged conversation and new ways of thinking about healthy lifestyle changes and ways communities can change together through media support:

*… Zoom class … a Somali exercise group during the lockdown. There is a health visitor, a dietitian… would come in those weekly meetings, and (ask) what have you guys done during the lockdown, what kind of exercise, and they were sharing stories … and then there will be that healthy conversation.”—**Somali***

Active collaborations between communities, health providers and experts had different formats but often involved having virtual community meetings to explain the government and health service perceptions and messages. For instance, in the Asian and Somali communities, religious leaders and centres collaborated with the local Government and NHS to create culturally relevant resources based on official government information, whilst African-Caribbean participants indicated that in their communities, churches and their leadership circulated information on preventing COVID-19 across their communities. Some ethnic minority health professionals also directly interacted with community members. For instance, the African-Caribbean community leaders invited a Director of Public Health, who was of African-Caribbean heritage, to speak to the Black community. These types of communication channels created a non-traditional route for sharing government information. In addition, participants stated that these sessions were beneficial in providing a safe space for community members to share their concerns about COVID-19 and be informed by trusted professionals from and within their community. Furthermore, these information avenues had a broader impact as the information was circulated by community members whom the groups trusted:

*… Somali doctor that lives in Leicester … speaking to our community and she had to make a video … (with) Somali subtitle as well, because people can actually read it … about trying to explain the vaccine, saying that I have taken it, you guys can make up your own mind, but it is safe, it’s for our health.—**Somali***

*We use our own initiative to develop videos in different languages. We produce leaflets, we did leaflet drops … to engage with the community … it’s been kind of drip feed to the local authority, NHS organisation public health, which we have tapped into, but we simplify things to ensure that the message is more appropriate for that particular community … help others, make them understand the severity of COVID impact on them.—**South Asian***

*… very real thing hearing it from somebody who you presumed to be human rather than somebody who is a professor … assisted a lot of us in coming to grips with some of the myths … they needed somebody to say that.—**African-Caribbean***

### 3.4. Community Recommendations to Improve Primary Care Communication

Participants suggested actions that should be implemented to improve healthcare communication. They stated that the lessons learned during the pandemic were very crucial and should not be ignored. Instead, proven effective interventions implemented at the public level and through primary healthcare should be embedded into the health services. These included improving communication and conversations based on shared values and GPs being available to talk informally through platforms such as community radios and virtual public meetings.

A summary of the recommendations is outlined in Table 2, with supporting quotes to provide context for the suggestions.

## 4. Discussion

This qualitative study explored the health communication and the access of ethnic communities in Leicester, United Kingdom, to primary healthcare services during the COVID-19 pandemic. Differences between minority ethnic groups were not distinct, and variations in experiences between the three ethnic groups could not be distinguished. The word cloud presented the participants’ commonly used words, summarising the key areas of emphasis: knowledge, community, information, COVID, the government, trust, sharing, family, social and health issues. Sources of health information varied, but community representatives stated that shared information was often inconsistent, prompting high distrust. Furthermore, the evidence highlighted that the health system and providers ignored cultural factors when communicating with patients from ethnic minority backgrounds. These neglected issues, and information circulation among the community, instigated other fears and gave rise to conflicting information and confusion, especially on the issue of vaccination, consequently making ethnic minorities vulnerable and often reliant on mis- and dis- information. The health outcomes and service accessibility experiences of the communities had both direct and indirect impacts, affecting households and communities as a group, especially large households, which was mostly common among South Asian communities. To manage the gap in communication and access, community organisations took up active roles to support their people, and recommendations for future healthcare plans and implementation include using the lessons from the pandemic improvement, including community leadership which would ensure culturally appropriate interventions are implemented and in turn improve trust in government actions.

Similar health communication sources and preferences during the pandemic were observed across different countries and age groups [3,27,28,29]. However, for ethnic minorities, tailored health messages shared through culturally accessible delivery modes, such as religious leaders, were reported to be more effective in reaching the intended communities [30]. This approach ensured that socio-cultural factors such as generational differences, religious beliefs and language preferences were adequately considered, as critically highlighted in this study. In addition, sub-groups within communities, such as the elderly, may need to be considered separately as acceptance of public health messages is often affected by factors such as age [30], and this was highlighted in this study in the reluctance of elderly populations to accept information explained by younger people. Language, cultural understanding and competence used in health services significantly influence the way patients access care, especially in ethnic minority populations, where language barriers have been reported as the dominant factor affecting healthcare access [5]. Therefore, using language and context that reflect ethnic minority social-cultural norms and relevant socio-demographics often increases motivation, acceptance and engagement [31]. In the process, this could minimise fear and increase trust in health information shared by authorities.

During the pandemic, confusion from mixed COVID-19 messages, different national guidelines, and the uncertainty of where to access relevant information raised skepticism and eroded the trust in the government and authorities [32,33,34]. This uncertainty causing increased anxiety and stress, particularly related to health and economic fears, has also been observed in other countries [35,36,37]. For instance, a study in Singapore showed common responses to conflicting COVID-19 messages included fear, concern, panic buying and hoarding [35]. Such reactions show the public’s frustration and anger, leading people to take actions into their own hands, including seeking information from other sources such as from their countries of origin, internationally, and has been commonly observed among migrant communities [38]. Similar to other countries, levels of trust in information provided through government sources had a significant influence on the actions of people, including COVID-19 vaccine uptake [33,35,38,39]. For example, a study from the US reported that people who relied on “conservative” news outlets and who had low confidence in scientists were least likely to vaccinate, and this was higher amongst ethnic minorities [37]. Such mistrust in government sources increased fears and exposure to multiple information sources, including social media, and this often opened them up to mis-/disinformation, including conspiracy theories [35,40]. A previous study observed that COVID-19 conspiracy beliefs were more widespread in countries with a polarised political and media environment [27]. A consequence of this was shown in a similar study conducted in a UK multi-ethnic setting, where it was reported that during the pandemic, the more confused, distressed and mistrusting people felt, the less positive they were about a vaccine, and this was particularly heightened within marginalised groups who had pre-existing reasons for mistrusting institutions, e.g., ethnic minority groups [3]. The same study also shared that local and targeted responses, such as using local leaders and providing accessible COVID-19 information on local government websites, helped reduce misinformation and improve trust [3]. Delivering health messages via trusted sources can increase message persuasiveness [41] and minimise belief in conspiracy theories and myths, which are barriers to complying with guidelines [25,32]. Furthermore, health messages conveyed via local, trusted sources are important as people are more likely to engage with guidance when the message comes from within their community [42,43]. These observations highlighted the significant role community leaders could play in supporting local initiatives and health communication acceptance in communities, predominantly ethnic minority communities. Overall, it has been suggested that improving health messaging to increase adherence to public health measures and behaviours such as attitudes to reduce COVID-19 transmission requires emphases on clarity of information, timely updates, clear communication between the government and the public, culturally and demographically appropriate delivery of positively framed public health advice, and adequate support measures [33,34,35]. However, one study in the UK reported that positively framed vaccine information delivered through government sources also heightened suspicions and mistrust due to concerns about the lack of transparency of risks [44]. Hence, clarity and transparency of information can be considered most important in building communication trust.

The rapid changes in the COVID-19 response profoundly affected primary care delivery. Care was delivered primarily via telephone, email, and video consultations [45,46]. Clinical triaging was actively implemented by using the NHS 111 online and non-clinical telephone triage service. As 111 staff focused on acute COVID-19 symptoms, patients were often denied the opportunity to discuss long-term care options for COVID-19 and other serious conditions with familiar healthcare professionals [47]. This allowed continuity of care but meant that only selected patients received face-to-face consultations, and most were unsatisfied with this approach. A study on restricted healthcare access during the COVID-19 pandemic also expressed concern about this healthcare approach [48]. The paper highlighted that older adults and vulnerable patients were held to the same national COVID-19 clinical pathway despite the higher risk and mortality rate among those groups; hence, patients often deteriorated before being provided care, which was sometimes neither effective nor efficient [48]. From the perspective of participants in this study, this care pathway was insufficient, and many felt that face-to-face was preferable to video and pictures for patients.

One critical point emerging from the study was the need to ensure that the lessons learned from the pandemic experience are used to improve future healthcare services. One core area highlighted is the need for increased support in mental health. A study on implementing remote consulting in UK primary care following the COVID-19 pandemic reported that consultation rates in patients with poor mental health and shielding patients increased [49]. For children and adolescents, the social restrictions—use of social distancing and stay-at-home orders—significantly impacted their mental and emotional wellbeing [50]. An example is shown in a study in Ireland that reported increased feelings of social isolation, depression, anxiety, despair, and maladaptive behaviour among children, especially among those with underlying behavioural conditions such as autism spectrum disorders who experienced severe routine disruption [50]. These findings are of particular concern and can have long-term impacts, as the emotional consequences of this increased risk of psychiatric disorder experiences, such as depression and anxiety, can contribute to adverse psychological experiences in childhood, which have been shown to be associated with an increased risk of anxiety later in life [51,52]. As such, young people’s mental health needs priority consideration and support systems to mitigate any long-lasting psychosocial effects from the COVID-19 pandemic.

Several reports discovered that exposure to traditional media was strongly and negatively associated with anxiety and depression and that exposure to digital media and personal contacts was positively associated with anxiety and depression, and less likelihood of taking up vaccination [27,37]. Hence, to manage anxiety during the pandemic, some participants decided to limit their intake of COVID-19-related news because it was too distressing. This finding was similar to another multi-ethnic study where it was also reported that exposure to health experts rather than politicians was another way of managing anxiety [3]. However, with poor acknowledgement of mental health among ethnic minority populations, the levels of adverse mental health within communities have yet to be determined. Therefore, more awareness is needed so the effects of extended social isolation, especially among vulnerable individuals, can be identified and managed, especially in ethnic minority communities. An excellent example of learning to adopt methods from the pandemic is the continuous mobilisation of pre-existing partnerships and community health ambassadors providing insight into community needs.

The social impacts, such as economic constraints and family tensions highlighted in the research, were shared across all the ethnic groups that participated in the study. However, group variations were primarily related to interpersonal and cultural dynamics and beliefs. Examples can be observed in the relationship between elderly family members and their younger familial support systems. South Asian ethnic groups were much more likely to live in larger and multi-generational households [7,8], and this sometimes caused communication friction, especially between different generations. Hence, although all groups confirmed the shared role in supporting elderly family members, South Asian representatives also explained that the generational friction between the ages was due to the cultural practices of filial piety, which prioritise the perspectives of the elderly while disregarding the input of younger family members. This main difference between ethnic groups and their shared experiences of marginalisation within broader society makes minority groups unique and fertile ground for additional exploration.

The underlining theme in this research elaborates on the importance of clear communication with ethnic minority communities which could apply to other vulnerable groups, and the need to better understand how to support them, especially in times of crisis. To achieve this, the communication pathways, cultural needs, and bridging of language barriers need to be taken into consideration [10,38,53]. A core component observed from all the communities in this study needed for strengthening related interventions would be the involvement of communities in developing communication materials and strategies. Working with ethnic minority groups to produce appropriate communication encourages acceptance and increases adherence, including increasing vaccine uptake [10]. Hence, communication pathways for minority groups need to include community contributions—as both providers and recipients of the information. This two-sided involvement would ensure that cultural needs and language issues are addressed in the information development and communication delivery. This would increase accessibility to accurate information, acceptance and adherence, and help combat mis-/disinformation, conspiracy theories and anti-vaccination campaigns. In addition, improving communication between healthcare providers and patients is important; this includes improving healthcare provider communication skills by providing them with training on cultural diversity sensitivity, stereotyping and prejudice, and specific skills to negotiate communication barriers [10,54]. Such skills will be particularly helpful when communicating with elderly ethnic minority members [54].

### 4.1. Strength and Limitation

This study presents a qualitative account of ethnic minority populations’ health communication and primary healthcare interactions during the COVID-19 pandemic. The main strength of the study is the novel insights gained from different participant perspectives, which provide a rounded account of ethnic minority populations’ communication and primary healthcare experiences. Thus, this study primarily explored the social factors that contributed to the sharing of healthcare communications in ethnic minority communities in a specific location. As a result, the research provides a view of the various relationships that influence community member actions, perceptions of local authorities in community engagement and the way cultural practices shape social dynamics. This qualitative approach contributes to previous research findings that have examined COVID-19 experiences across multiple socio-demographic factors and levels. The disaggregated social factors that shaped ethnic minority relationships in communities and the way experiences shaped information acceptance during the pandemic are also reflective of other multi-ethnic locations.

However, since the research was conducted in only one location with specific populations, it may not be widely generalisable. In addition, not all participants were willing to share their demographic information, such as age, so further socio-demographic analysis beyond ethnicity could not be carried out. In addition, the relationships with local authorities, community-led initiatives, and support systems may vary according to location. Hence, future research is needed to explore how local authorities engage, support and empower minority communities. Limited ethnic representation in this research restricted in-depth exploration of experiences from more ethnic groups and healthcare services; hence, the results may not adequately reflect all ethnic minority populations or healthcare services. As such, more research is required to broaden the minority population groups, for instance, Gypsy Traveller and Eastern European communities. A broader outlook of the communication dynamics from different minority groups will allow for in-depth analyses of the way cultural practices influence relationships with local authorities, healthcare providers and interpersonal relationships.

Due to the short duration of the study, only a small number of interviews and FGDs were conducted; nevertheless, data saturation was reached on the topics explored. In future studies, where possible, FGD participants or sessions can be increased by extending the length of the study and expanding the data collection methods to allow opportunities for exploring and following up on critical issues, such as the long-term impacts. In addition, the experiences of ethnic minorities during the pandemic, pressure and frustration with situations may have influenced their responses. Hence, potential follow-up research could revisit some of the emerging themes from this research to determine areas of change in interpersonal and community-based communication and relationships between local authorities and the community. These findings will also allow policy makers and holders to assess the viability of interventions and support implemented during the pandemic and their immediate aftermath.

### 4.2. Implications for Research and Practice

Ethnic minorities have been disproportionately affected by the COVID-19 pandemic due to a complex interplay of factors, including cultural factors and access inequalities. Reducing these inequalities requires a greater understanding of the related factors, which include health communication. Primary care, which is the first point in healthcare services, has to meet the needs of the whole population, and this includes understanding the socio-demography and unique socio-cultural factors that influence access to care for all patients, including ethnic minority communities.

Key suggestions for improving health communication and minimising primary access inequalities include the need for local health authorities to continue working with community leaders, maintain a presence and support ethnic minorities; co-production of health messages with community members; provision of adequate guidance and information in multiple relevant languages, and the use of formally trained interpretation options in triage services. There also needs to be an acknowledgement of the factors that influence the way patients interact with information, which could be an area for future research and action to explore the multi-dimension and complex interplay between ethnicity, health access and other inequalities. Hence, communication pathways for minority populations need to include members of those communities in the development and delivery of information to improve accessibility, acceptance and adherence to public health advice.

## 5. Conclusions

Varied communication sources and preferences were adopted among ethnic minority communities, and primary healthcare services were limited and mostly digitised. However, a significant challenge to accessibility, communication and primary care service was language barriers and poor access/use of digital technology, and these factors mostly affected the elderly populations. In addition, delay, lack of clarity and inconsistency in health information limited the trust of communities in government and health providers, making ethnic minorities vulnerable to mis-/disinformation. Therefore, in order to improve future health communication strategies in primary health care and other health services, experiences from the pandemic should be reflected upon, and positive initiatives such as the use of community-tailored communication and health access support could be infused into the population and private healthcare strategies, especially for ethnic minorities.

## Figures and Tables

**Table 1:** Summary of identified themes and subthemes.

Theme	Sub-Theme
Health communication	Communication source and preference Vaccination communication Language barrier Community-tailored communication
Health experience, impact and management	General health experiences Caring responsibilities Mental health
Health services and management	Health services Community actions
Community recommendations to improve primary care communication	Learning from the pandemic Communication improvement Community leadership Cultural appropriate interventions Trust and government actions

**Table 2:** Recommendations for health communication and engagement improvement from community members.

Theme	Recommendation	Supporting Quote
Learning from the pandemic	Reflect on the entire pandemic experience step-by-step and identify the issues and what actions were used to tackle them for future preparedness	*… the lessons are only going to be learned if we can sit back, go through the whole process from start to the end … (identify), what were the issues coming from the committee itself. And how they have tackled issues step by step …—**South Asian***
	Need for more support structures, especially mental health support for children	*… mental health and wellbeing and it’s not just for adults, but also for young people or for children. There’s lot more where that needs to be done… from local government, or even from the government itself, providing support for people who suffer from ill mental health, mental wellbeing.—**African-Caribbean***
	More decision-making power from the national government should be provided to local authorities for easier and more effective management of community-related issues	*… (national) government is in charge of everything … afterwards giving a little authority to local councils, to take part in making decisions as well. Really speaking, it should be them (local councils) who should be taking control first and forward that information to the government … people have trust in them (local councils) more because they are dealing with local government in everyday life.—**South Asian***
	Provide intervention and support for vulnerable individuals and carers based on the experiences from the failure to do so at the early stages of the pandemic	*… not just in UK, but internationally. Early intervention and supporting individuals who were vulnerable, would be more crucial, especially when you look at some of the examples that residential homes where people were discharged from hospitals without testing going back into hospital… (carers) unpaid key workers, support them appropriately whether its health or social care.—**South Asian***
Communication improvement	Identify trusted sources from within the community to serve as representatives of health services and to share necessary information	*So to have a communication, trusted communication … where people of Caribbean, Black any heritage, but focus on that…communication of information, which seems a bit tricky … But the certain phrases, which seem to put people’s back up, we’ve got to be mindful, and perhaps having people engaging in the language would be, make people engage a bit better.—**African-Caribbean***
	Take advantage of the shift to do more digital communication and use those technologies to make information accessible to everyone	*…. I don’t need to be out on the road every day and I can be in contact with people all over the world. Because technology has shown me I can access conversations, discussions, information with pioneers in their field… I want that to be accessible for everybody. Not just for those who can afford it.—**African-Caribbean***
	Need for clearer, more accurate and consistent health messaging and limit mixed messages	*… if they give us clear messages rather than confusing … (and) share with the community one that is helpful … (authority) do their homework correctly. Then just give the right message and one message and stick to that. If you change it, give the reason for doing it rather than “shall we do this or not”.—**South Asian***
	Need for simplified and accessible information tailored to different demographic groups to minimize high reliance on others	*they want somebody who’s going to give them accurate information, its about how they put it across, and the medium that they use and put it across … it’s been primarily people in the mid to late 40s upwards … people in their 50s, they’re still all on Facebook, and other types of social media, and they’re still in the chat groups, and things get bounced around in that way.—**African-Caribbean***
Community leadership	Be aware and acknowledge intersections within the community towards influencing their decision-making and action	*… acknowledge the intersections of communities, because a lot of this ends up treating things like, Oh, so you’re African-Caribbean. But, like, I’m also part of many other communities … if you’re not supporting people through it all, not listening to them, not showing that you actually care about them as human beings, we’re not going to leave this pandemic.—**African-Caribbean***
	Use more community structures to deliver health communication and information	*… more community based, leading … the area I grew up in, they closed our library, and everyone complained that there were kids on the street … Seeing communities as communities again.—**African-Caribbean***
	Proper selection of ethnic minority representatives who are truly respected and acknowledged amongst their communities	*… not just to pick up somebody from the community itself to put on the decision-making process, you have to look into their background, and also whether it is acceptable to the committee or not … that person’s views are about equality and diversity, whether that person understands it.—**South Asian***
	Use trusted community members and sources to actively promote and advocate for improvement in vaccine uptake	*… having someone trusted within the community, not necessarily just having it … but speaking about it, acknowledging that there are concerns… and weighing up (what) seems to have a better impact.—**Somali***
Cultural appropriate interventions	Improve investment funding and establish policies for community health and wellbeing initiatives in different settings	*The government should invest more in health … in how people live a healthier lifestyle, if necessary, then some things should be imposed. I work in a school, I don’t think the kids are as healthy as they should … one of the reasons that we’ve been hit badly with COVID is that we have a big number of people who are unhealthy … So they have to do something about that.—**African-Caribbean***
	More government investment in health services to build more hospitals rather than close down hospital wards	*… financial assistance should be the first priority resource … that goes with the health services and build more hospitals … rather than just finding more, closing some wards and suddenly they had to open all this …—**South Asian***
	Invest in infrastructure for younger generations that reflect their culture	*… we used to have Grandby Halls and we used to meet friends … We’re still friends today … My daughter won’t have that. There is nowhere for them to go … Our children and grandchildren won’t have that opportunity unless we make it for them like that; the African-Caribbean centre is not run by African-Caribbeans, it is owned by the Council … they’ve got a few Asian faces, they think that’s enough representation, but they don’t represent us.—**African-Caribbean***
	Care for elderly people should appropriately take into consideration their cultural values and preference	*Our lovely elderly relatives to have care homes which acknowledge doing your hair is different … Sometimes the diets are different … have Christian ones … Muslim care homes … Mason’s, it’d be really wonderful to have one or two, which acknowledged that and actually catered …—**African-Caribbean***
	Local authorities should empower and support community organisations that engage and support general ethnic minority communities	*… local authority to give support to organisations like us (community organisation) … even to the nation, we helped … service that complements whatever they’re doing … many people will have been hospitalised … we prevented all this by providing this information and the right support … (important) the grassroot organisations have the support and the trust of the people—**Somali***
Trust and government actions	Provide timely and clear ethnic minority information, for instance, explanations of why more ethnic minorities were affected	*I would like to see why so many ethnic minority groups have died during COVID-19 … government becomes organised … it’s important that people get that trust, that people are honest … I see people who were victims of COVID-19, I see people who died from COVID-19 and the people lost so many loved ones, so I would like to know why.—**Somali***
	Authorities need to listen to the people’s opinions	*… if the government listened to the people, to us, our struggles, and how we’re dealing with it, then we’re gonna get through it.—**Somali***
	Government and health services need to be honest and transparent, present accurate information to encourage public cooperation	*So if the government show us the right figures, what they are doing at each stage, and not just suddenly change the rules without telling us why recently … we will be patient with them if they’re honest … They can’t expect us to do something that they’re not doing. So if they show us better examples, we will respect them more, we will listen …—**South Asian***
	Authorities should not mix politics and health issues but need to prioritise the health of people and focus on facts to improve public trust	*… government need to separate politics from the health of the people. You cannot play with the politics in health. Health has to be the priority … People are not robots … trust building will take time and has to be nurtured … politicians have to be also accountable to the rules …—**Somali***
	Government authorities should avoid exaggerating health communication and reassure people with clear supporting information	*… (government) exaggerated a lot of things … they should have reassured people, yes, they did at the end. That’s not the point. You should reassure people … Rather than scaring people … people felt like they were left to their own measures … because you’ve not made it clear.—**Somali***

## Data Availability

The data presented in this study are available on request from the corresponding author. The data are not publicly available due to data protection policy and participant requests.

## Supplementary Materials

The following supporting information can be downloaded at: https://www.mdpi.com/article/10.3390/ijerph192215166/s1, Supplementary S1: Research Topic Guide.
