# Supplementary material for: Health Communication and Inequalities in Primary Care Access during the COVID-19 Pandemic among Ethnic Minorities in the United Kingdom: Lived Experiences and Recommendations
Source: Int J Environ Res Public Health. 2022 Nov 17;19(22):15166. doi: 10.3390/ijerph192215166 (PMC9690007; doi:10.3390/ijerph192215166)
Supplement: Supplementary file 1 [file ijerph-19-15166-s001.zip › ijerph-2011010-supplementary.pdf]

UNIVERSITY OF LEICESTER

Centre for BME Health East Midlands

Topic Guide

Title: Covid-19 Information Flow, Health Inequalities and Public Health Impact among Ethnic Minority Communities in Leicester

Introduction

- Can I ask to confirm that you are happy to take part in this interview and for it to be recorded to be transcribed by a researcher at a later date?
- The aim of this focus group/interview is to find out how you have been coping with the pandemic and the sources and methods you have used to obtain pandemic related information.
- You are free to say as much as or as little as you wish in response to questions.
- You can stop the interview at any time.

Intro

- Have you or a member of your family been affected by COVID-19?
- How have you been coping with the pandemic?
- What communication sources and methods have you used to obtain pandemic related information?
- What types of information and messages have you received about the pandemic?

Giving results

- What did you think about the information you heard/received?
  Prompt: Was it in a way that you understood?

- Could anything have been made the information clearer?
  Prompt: Health risks, health outcomes, vaccines, location, time, people group, language

Follow up

- Did you speak to any health personnel (including a GP) about the health-related information you received?
- Did you speak to or share with anyone else in your community the information you received?
- What other information would you need to manage the pandemic better?
- Would you have liked any more support from the information source?
